# Sonic Hedgehog Signaling Inhibition Provides Opportunities for Targeted Therapy by Sulforaphane in Regulating Pancreatic Cancer Stem Cell Self-Renewal

**DOI:** 10.1371/journal.pone.0046083

**Published:** 2012-09-28

**Authors:** Mariana Rodova, Junsheng Fu, Dara Nall Watkins, Rakesh K. Srivastava, Sharmila Shankar

**Affiliations:** 1 Department of Pathology and Laboratory Medicine, The University of Kansas Cancer Center, The University of Kansas Medical Center, Kansas City, Kansas, United States of America; 2 Department of Pharmacology, Toxicology and Therapeutics, and Medicine, The University of Kansas Cancer Center, The University of Kansas Medical Center, Kansas City, Kansas, United States of America; Indiana University School of Medicine, United States of America

## Abstract

Dysregulation of the sonic hedgehog (Shh) signaling pathway has been associated with cancer stem cells (CSC) and implicated in the initiation of pancreatic cancer. Pancreatic CSCs are rare tumor cells characterized by their ability to self-renew, and are responsible for tumor recurrence accompanied by resistance to current therapies. The lethality of these incurable, aggressive and invasive pancreatic tumors remains a daunting clinical challenge. Thus, the objective of this study was to investigate the role of Shh pathway in pancreatic cancer and to examine the molecular mechanisms by which sulforaphane (SFN), an active compound in cruciferous vegetables, inhibits self-renewal capacity of human pancreatic CSCs. Interestingly, we demonstrate here that Shh pathway is highly activated in pancreatic CSCs and plays important role in maintaining stemness by regulating the expression of stemness genes. Given the requirement for Hedgehog in pancreatic cancer, we investigated whether hedgehog blockade by SFN could target the stem cell population in pancreatic cancer. In an *in vitro* model, human pancreatic CSCs derived spheres were significantly inhibited on treatment with SFN, suggesting the clonogenic depletion of the CSCs. Interestingly, SFN inhibited the components of Shh pathway and Gli transcriptional activity. Interference of Shh-Gli signaling significantly blocked SFN-induced inhibitory effects demonstrating the requirement of an active pathway for the growth of pancreatic CSCs. SFN also inhibited downstream targets of Gli transcription by suppressing the expression of pluripotency maintaining factors (Nanog and Oct-4) as well as PDGFRα and Cyclin D1. Furthermore, SFN induced apoptosis by inhibition of BCL-2 and activation of caspases. Our data reveal the essential role of Shh-Gli signaling in controlling the characteristics of pancreatic CSCs. We propose that pancreatic cancer preventative effects of SFN may result from inhibition of the Shh pathway. Thus Sulforaphane potentially represents an inexpensive, safe and effective alternative for the management of pancreatic cancer.

## Introduction

Pancreatic cancer (PC) has one of the poorest prognoses among all cancers and overall 5-year survival rate of 3% [1], [2], [3]. Unfortunately, in most cases pancreatic cancer is not resectable at the time of diagnosis. There are limited treatment options available for this disease because chemo- and radio-therapies are largely ineffective, and metastatic disease frequently redevelops even after surgery [4]. Therefore, there is an urgent need to discover novel and effective chemopreventive approaches for pancreatic cancer.

Cancer stem cells/tumor initiating cells (TICs) have been proposed to be the cause of cancer initiation, progression and chemotherapy failure in several human malignancies including pancreatic cancer [5], [6], [7]. The CSC hypothesis suggests that only the stem cell compartment in tumors is capable of unlimited self-renewal and that elimination of these cells will ultimately halt neoplastic expansion, as better-differentiated cells have limited mitogenic capacity and will not contribute to long-term tumor growth. Therefore, it is imperative to design new strategies based upon a better understanding of the signaling pathways that control aspects of self-renewal and survival in CSCs in order to identify novel therapeutic targets in these cells. Thus, development of therapeutic strategies that specifically target pancreatic CSCs can be effective in eradicating tumors and in reducing the risk of relapse and metastasis.

Upregulation of sonic hedgehog pathway have been demonstrated in CSCs, which effect tumor progression including migration, invasion and metastasis. Inappropriate activity of the Hh signaling pathway also has been linked to tumor types that arise sporadically or in genetically predisposed individuals [8], [9]. The Shh pathway is an early and late mediator of tumorigenesis in epithelial cancers. Activation of Shh signaling seems to precede transformation of pancreatic tissue stem cells to pancreatic cancerous stem cells, with Gli transcription factor functioning as a mediator of environmental signals and in the progression of pancreatic CSCs into metastatic tumor cells. Shh signaling is launched by binding of the secreted Shh peptide to the 12- span transmembrane protein Patched (Ptch), resulting in loss of Ptch activity and consequent phosphorylation and posttranscriptional stabilization of 7-span transmembrane protein Smoothened (Smo), a member of the serpentine receptors [10], [11]. As a result, expression of Hh target genes is initialized through posttranslational activation of the Gli family of zinc-finger transcription factors [12]. The Gli family is one of the target gene consistently induced whenever the Shh pathway is activated, making this transcript a reliable marker of both physiologic and pathologic Shh signaling activity. Activation of Shh signaling pathway is involved in the regulation of the proliferation of the pancreatic CSCs. Thus by targeting signaling pathways that are aberrantly activated and of importance for the maintenance of cancer stem cell may lead to the development of novel treatment regimens for pancreatic cancer by the elimination of pancreatic cancer CSCs [13], [14].

Epidemiological studies have suggested that increased risks of pancreatic cancer are associated with tobacco, obesity and high consumption of fat, fish, pork or beef, and that decreased risks are associated with consumption of cruciferous vegetables. An important group of agents that have this property are the organosulfur compounds such as isothiocyanates (ITCs), abundant in cruciferous vegetables for which consumption has epidemiologically shown an inverse link with pancreatic cancer. ITCs have been shown to exhibit several potential chemoprotective activities in cell and animal models [15], [16], [17], [18]. We have previously shown that oral administration of sulforaphane inhibited the growth of PC-3 cells orthotopically implanted in the prostate of nude mice by inducing apoptosis and inhibiting tumor cell proliferation, metastasis and angiogenesis [19]. We have also recently demonstrated that SFN alone and in combination with quercetin inhibits growth of pancreatic cancer stem cells derived from pancreatic cancer cell lines *in vitro* through the regulation of FOXO proteins [20]. Thus, SFN holds great promise for development as a chemopreventive/therapeutic agent. In spite of these finding, there are no studies demonstrating the regulation of pancreatic CSCs by SFN, and whether SFN can inhibit Shh pathway.

Thus the objective of this study was to investigate the role of Sonic hedgehog pathway in pancreatic cancer and to examine the molecular mechanisms by which sulforaphane (SFN), an active compound in cruciferous vegetables, inhibits self-renewal capacity of pancreatic CSCs. The underlined molecular mechanism in exhibiting this effect was through inhibition of Sonic hedgehog signaling pathway at the level of Gli transcription, proliferation, stem cell characteristics and induction of apoptosis. These data suggest that SFN can be a beneficial agent for the prevention and treatment of pancreatic cancer. Such information will not only allow rational design of SFN-based strategies for prevention and/or treatment of pancreatic cancer but could also facilitate development of mechanism-driven protocols for optimal clinical effects.

## Results

### Expression of sonic hedgehog signaling pathway genes in pancreatic CSCs

**Figure 1:**
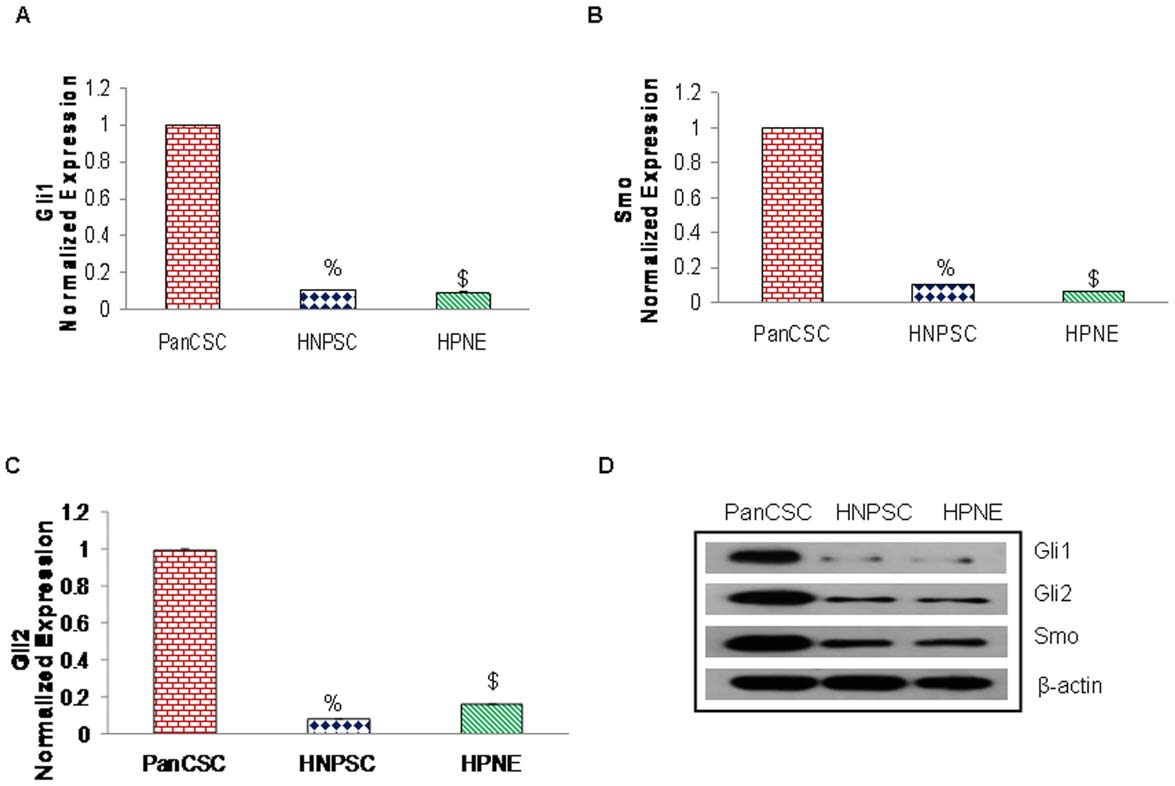
Expression of Shh pathway in pancreatic CSCs. (**A–C**), Relative expression of various components of Shh pathway was quantified in human pancreatic cancer stem cells (PanCSC), human pancreatic normal stem cells (HNPSC) and human pancreatic normal ductal epithelial cells (HPNE). The expression of Shh genes was quantified using quantitative reverse transcriptase polymerase chain reaction real-time assay (q-RT-PCR), and normalized to GAPDH expression. All assays were performed in triplicate and were calculated on the basis of ΔΔ*C*t method. Data represent mean ± SD. %, and $ = significantly different from control, P < 0.05. (**D**), Immunoblotting of Gli1/2, Smo and β-actin of human pancreatic cancer stem cells (PanCSC), human pancreatic normal stem cells (HNPSC) and human pancreatic normal ductal epithelial cells (HPNE).

The Hedgehog (Hh) signaling pathway is essential for the development of tissues and organs [8]. However, aberrant activation of sonic hedgehog (Shh) signaling pathway plays important roles in tumorigenesis and progression of several tumors [8], [9]. To evaluate the possibility that the Hh signaling is active in human pancreatic CSCs we compared the mRNA expression of various components of the Shh pathway in human pancreatic CSC (PanCSC) to human pancreatic normal ductal epithelial cells (HPNE) and human normal pancreatic stem cells (HNPSC), in an *in vitro* cell culture model and as quantified by qRT-PCR. As shown in Fig. 1.A–C, we observed a robust expression of key components of the Shh-Gli pathway in pancreatic CSC as compared to their normal counter parts. The protein expression of these components was further confirmed by Western blotting as shown in Fig. 1.D. Interestingly, we demonstrate here that Sonic hedgehog pathway is highly activated in pancreatic CSCs suggesting that hyperactive Shh-Gli signaling may regulate the expression of stemness genes in pancreatic CSCs and play a role in the cell proliferation and progression of pancreatic CSCs.

### Sulforaphane inhibits hedgehog signaling pathway in pancreatic CSCs *in vitro*

**Figure 2:**
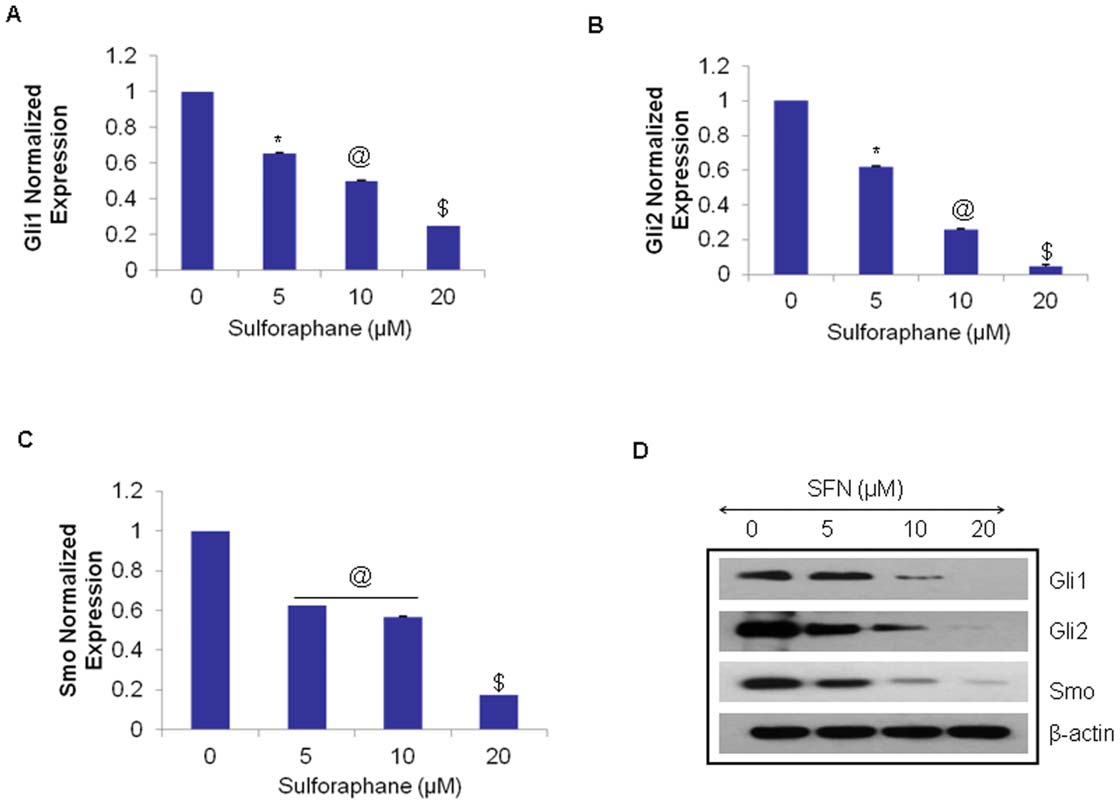
Regulation of Shh pathway by SFN in pancreatic CSCs *in vitro*. (**A–C**), Inhibition of components of sonic hedgehog pathway. Pancreatic CSCs were treated with sulforaphane (0–20 µM) for 24 h. The expression of Gli1, Gli2 and Smo was measured by qRT-PCR and normalized to GAPDH expression. All assays were performed in triplicate and were calculated on the basis of ΔΔ*C*t method. Data represent mean ± SD. *, @, and $ = significantly different from control, P < 0.05; (**D**), Immunoblotting of Gli1/2, Smo and β-actin of human pancreatic cancer stem cells (PanCSC) treated with Sulforaphane (0–20 µM).

**Figure 3:**
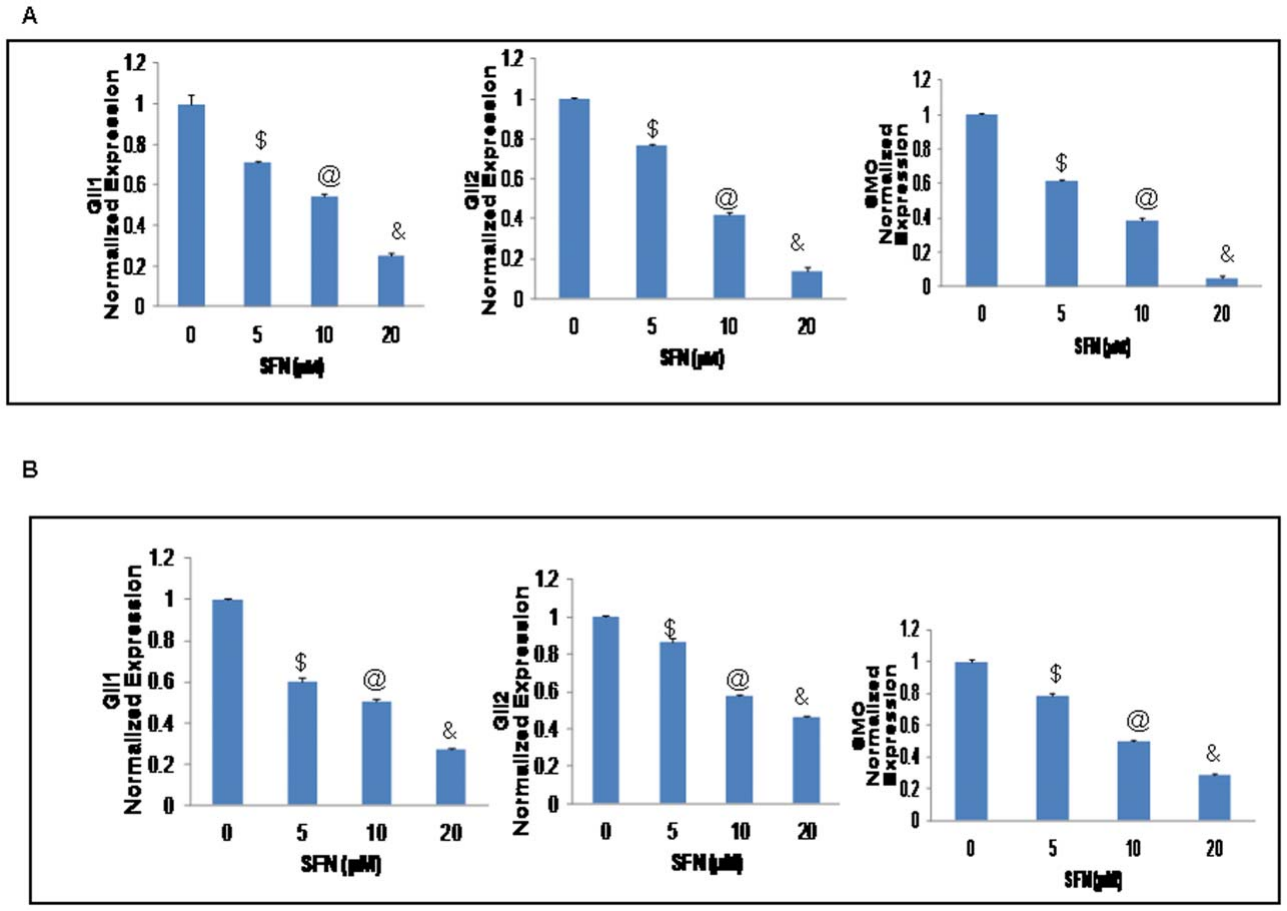
Regulation of Shh pathway by SFN in pancreatic cancer cell lines *in vitro*. (**A–B**), Inhibition of components of sonic hedgehog pathway. ASPC1 and PANC1 were treated with sulforaphane (0–20 µM) for 24 h. The expression of Gli1, Gli2 and Smo was measured by qRT-PCR and normalized to GAPDH expression. All assays were performed in triplicate and were calculated on the basis of ΔΔ*C*t method. Data represent mean ± SD. $, @, and & = significantly different from control, P < 0.05.

We therefore sought to examine the effects of SFN on the expression of Shh receptor (Smothened) and effectors (Gli1 and Gli2) by qRT-PCR in human pancreatic CSCs. As seen in (Fig. 2.A–C), SFN inhibited the expression of Smo, Gli1 and Gli2. Since we have previously shown the Shh pathway activation in a panel of human pancreatic cell lines [21], we also sought to examine the effects of SFN on the expression of Shh receptor (Smothened) and effectors (Gli1 and Gli2) by qRT-PCR in pancreatic cancer cells ASPC and PANC-1. As shown in Fig. 3, SFN inhibited the expression of Smo, Gli1 and Gli2 in these cell lines as well, suggesting that SFN can inhibit the activation of Shh signaling in pancreatic cancer.

**Figure 4:**
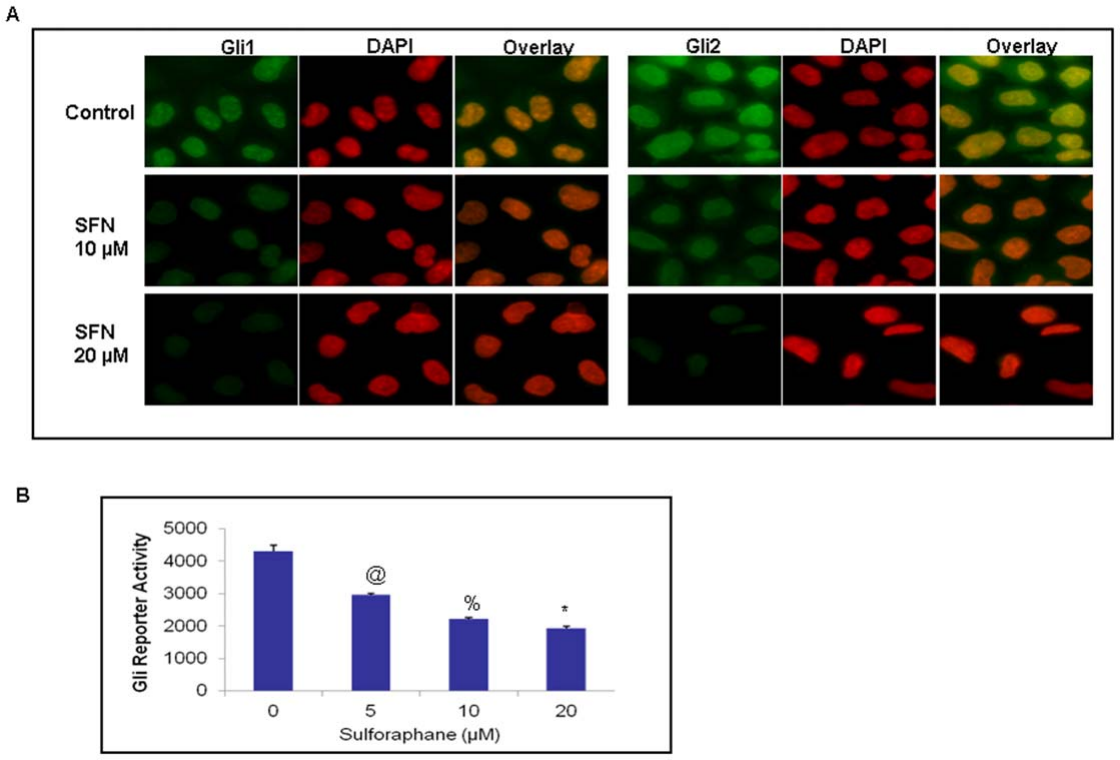
Effects of SFN on Gli translocation and transcription. (**A**), The nuclear translocation of Gli1 and Gli2, was measured by immunocytochemistry. Pancreatic CSCs were treated with sulforaphane (0–20 µM) for 24 h. Cells were then stained with anti-Gli and Gli2 antibody (green fluorescence), and DAPI (red fluorescence). Merged images are shown, which indicate yellow-orange staining of Gli 1 and Gli2 located in the nucleus due to co-localization of green and red fluorescence. (**B**), Inhibition of Gli transcription. Pancreatic CSCs were transduced with Gli-responsive GFP/firefly luciferase viral particles (pGreen Fire1-Gli with EF1, System Biosciences). After transduction, culture medium was replaced and CSCs were treated with sulforaphane (0–20 µM) for 24 h. Gli-responsive reporter activity was measured using a luciferase assay (Promega Corporation). Data represent mean ± SD. @, %, and * = significantly different from control, P < 0.05.

Further, since the presence of nuclear Gli expression is indicative of active Hh signaling, we examined the effects of SFN on the nuclear translocation of Gli transcription factors using immunocytochemistry (ICC). Pancreatic CSCs were treated with SFN (0–20 µM) for 24 h. Cells were then stained with anti-Gli and Gli2 antibody (green fluorescence), and DAPI (red fluorescence). Merged images are shown, which indicate yellow-orange staining of Gli 1 and Gli2 located in the nucleus due to merge of green and red fluorescence. As shown in Fig. 4A, SFN inhibited the nuclear translocation of Gli1 and Gli2, as measured by ICC. These data therefore, suggest that SFN inhibits sonic hedgehog pathway at the level of Gli activation.

### Disruption of Shh signaling by Sulforaphane inhibits Gli reporter activity in Pancreatic CSCs

Since Gli transcription factor mediates the effects of Shh which play important roles in maintaining stemness and tumorigenesis, we next measured the Gli transcriptional activity (Fig. 4B). Pancreatic CSCs were transduced with Gli-responsive GFP/firefly luciferase viral particles (pGreen Fire1-Gli with EF1, System Biosciences). SFN inhibited Gli transcriptional activity in a dose-dependent manner. These data suggest that SFN can regulate pancreatic carcinogenesis which is mediated through the inhibition of Shh signaling.

### Inhibition of Gli enhances the effects of sulforaphane on spheroid formation in Pancreatic CSCs

**Figure 5:**
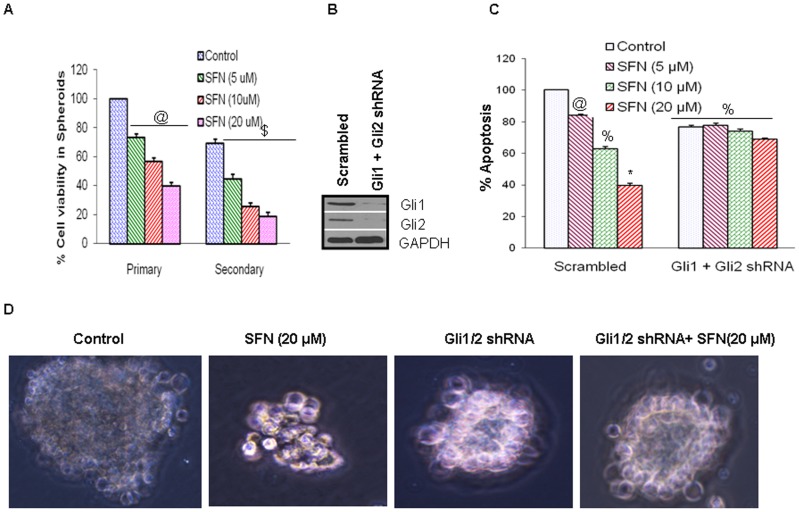
Regulation of Gli on spheroid formation by SFN in pancreatic CSCs *in vitro*. (**A**), Inhibitory effects of SFN on CSC's spheroid viability. Pancreatic CSCs were seeded as described above and treated with SFN (0 and 20 µM). After 7 and 14 days, primary and secondary spheroids were dissociated and viable cells were counted by trypan blue assay. Data represent mean ± SD. @, and $ = significantly different from control, P < 0.05. (**B**), Pancreatic CSCs were transduced with either scrambled shRNA or Gli1 and Gli2 shRNA expressing lentiviral vectors (pLKO.1), and cell lysates were collected and Western blot analysis was performed using anti-Gli1 or Gli2 antibody. (**C**), CSC/scrambled and CSC/Gli shRNA were seeded as described above and treated with SFN (0–20 µM). After 7 days, spheroids were collected and cell suspensions were prepared and viable cells were counted by trypan blue assay. Data represent mean ± SD. @, %, and * = significantly different from control, P < 0.05. (**D**), Gli shRNA enhances the inhibitory effects of SFN on CSC's spheroid viability. CSC/scrambled and CSC/Gli shRNA were seeded as described above and treated with SFN (0 and 20 µM). After 7 days, spheroids were photomicrographed.

Furthermore, to determine the effect of SFN on Shh signal inhibition on pancreatic CSC proliferation and self renewal, we performed sphere formation assay. Pancreatic CSCs were treated with SFN (0–20 µM) for 1 week and assessed for its intrinsic ability to form primary spheres in culture. At the end of one week, total numbers of spheres were measured and ability to form secondary spheres was further assessed for another week. Human pancreatic CSCs derived spheres were significantly inhibited on treatment with SFN, suggesting the clonogenic depletion of the CSCs (Fig. 5A).

Given the requirement for Hedgehog signaling in pancreatic cancer, we investigated whether Hedgehog blockade by sulforaphane could target the stem cell population in pancreatic cancer. To understand the role of Shh signaling in the SFN induced inhibition of pancreatic CSC proliferation, we sought to inhibit the Shh signaling in pancreatic CSCs by knocking down the Gli transcription factor using lentiviral mediated transduction of Gli shRNA inhibiting both Gli1 and 2 expression. The expression of Gli in pancreatic CSC was suppressed by nearly 100% using the Gli1 and Gli2 targeting shRNAs as quantified by Western blotting (Fig. 5B). As shown in (Fig. 5C–D), the percentage of sphere forming ability of pancreatic CSCs was significantly reduced in Gli knock-down cells relative to the control cells. Further, interference of Shh- Gli signaling through lentiviral – mediated silencing significantly blocked SFN induced inhibitory effects demonstrating the requirement of an active pathway for the growth of pancreatic cancer stem cells.

### SFN inhibits the expression of pluripotency maintaining transcription factors

**Figure 6:**
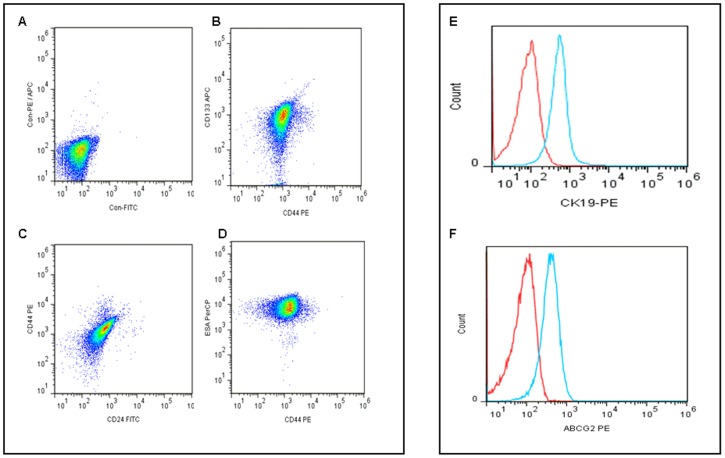
Characterization of human pancreatic CSCs from human primary tumors. (A–D), Expression of pancreatic stem cell markers. Flow cytometric analysis of Pancreatic CSCs expressing was performed using stem cell markers CD44 -PE, ESA-PerCP, CD133-APC, CD24-FITC and appropriate controls. (E–F), Expression of pancreatic epithelial markers and drug resistance genes. Flow cytometric analysis of Pancreatic CD44^+^ESA^+^CD133^+^CD24^+^ CSCs also expressed CK19-PE and ABCG2-PE respectively.

We have recently demonstrated that pancreatic CSC's expressing stem cells markers CD133, CD44, CD24, ESA, express high levels of pluripotency maintaining factors, and drug resistance genes MDR1 and ABCG2 as compared to normal pancreatic cells and pancreatic cancer cells [22]. We therefore characterized the CSCs isolated from human pancreatic tumors by flow cytometery used in the present study. As shown in (Fig. 6A–D), Pancreatic CSCs express stem cell markers CD44, ESA, CD133, and CD24. Interestingly, Pancreatic CD44^+^ESA^+^CD133^+^CD24^+^ CSCs also expressed CK19 (pancreatic cancer specific epithelial marker) and ABCG2 drug resistance genes (Fig. 6E–F).

**Figure 7:**
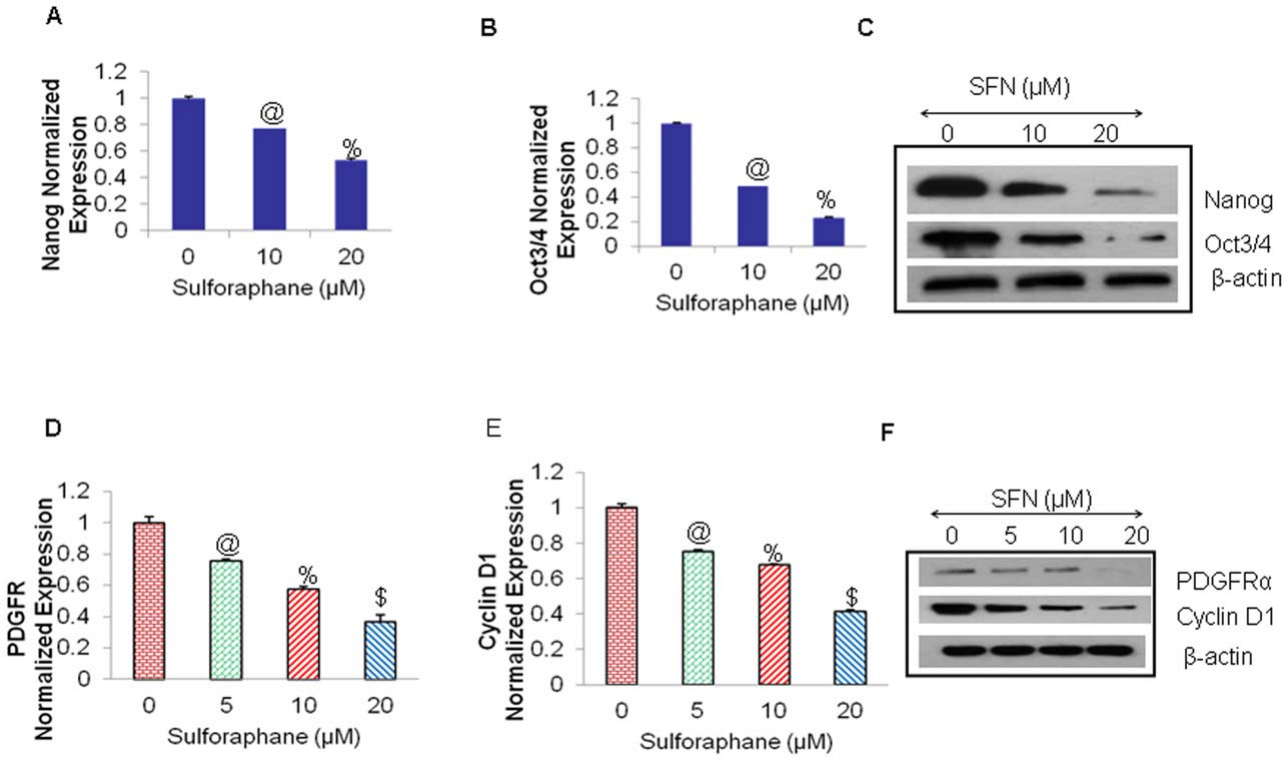
Regulation of Hh target genes involved in the maintenance of pluripotency in pancreatic cancer stem cells. (**A–B**), Effects of SFN on expression of Hh target genes in the pancreatic CSCs. Real time PCR (q-RT-PCR) was performed to examine the expression of Nanog and Oct4 and data were normalized with GAPDH. All assays were performed in triplicate and were calculated on the basis of ΔΔ*C*t method. Data represent mean ± SD. @ and % = significantly different from control, P < 0.05. (**C**), Pancreatic CSCs were treated with SFN (0–20 µM), and cell lysates were collected and Western blot analysis was performed using anti- Nanog, Oct4 or β-actin antibody. (**D–E**), Effects of SFN on expression of Hh target genes involved in cell proliferation in the pancreatic CSCs. Real time PCR (q-RT-PCR) was performed to examine the expression of PDGFRα and Cyclin D1, involved in the maintenance of proliferation was analyzed and normalized with GAPDH. All assays were performed in triplicate and were calculated on the basis of ΔΔ*C*t method. Data represent mean ± SD. @, %, and $ = significantly different from control, P < 0.05. (**F**), Pancreatic CSCs were treated with SFN (0–20 µM), and cell lysates were collected and Immunobloted for anti- PDGFRα, Cyclin D1 or β-actin antibody.

Nanog is essential for pluripotency, along with other ES like signature factors, such as OCT4 are also critical for pancreatic CSCs and other cancers [23], [24]. They are accepted Hh target genes as well. Indeed, the levels of OCT4 can induce epithelial dysplasia in mice, and has been implicated in various human tumors as also regulated by Hh-Gli signaling. We therefore, sought to examine the effects of SFN on the expression of these factors *in vitro*. As shown in (Fig. 7A–B), SFN inhibited the mRNA expression of Nanog, and Oct-4 in pancreatic CSCs. Further, as confirmed by Western blotting in (Fig. 7C), SFN inhibited the protein expression of Nanog, and Oct-4 in pancreatic CSCs. These data indicate that Shh signaling may promote pancreatic CSCs proliferation through enhanced cell self-renewal.

### Hedgehog signaling blockadge by sulforaphane inhibits the cell proliferation and induces apoptosis in Pancreatic CSCs

We next examined the effects of SFN on the mRNA expression of genes downstream of Gli transcription which are involved in cell proliferation. As shown in (Fig. 7D–E), SFN inhibited downstream targets of Gli transcription Cyclin D1 and PDGFRα in pancreatic CSCs which are known Hh target genes involved in proliferation. Further, the inhibition of protein expression of these targets by SFN in pancreatic CSCs was confirmed by Western blotting in (Fig. 7F). These data suggest that SFN can regulate pancreatic carcinogenesis by inhibiting Shh pathway and its downstream targets.

**Figure 8:**
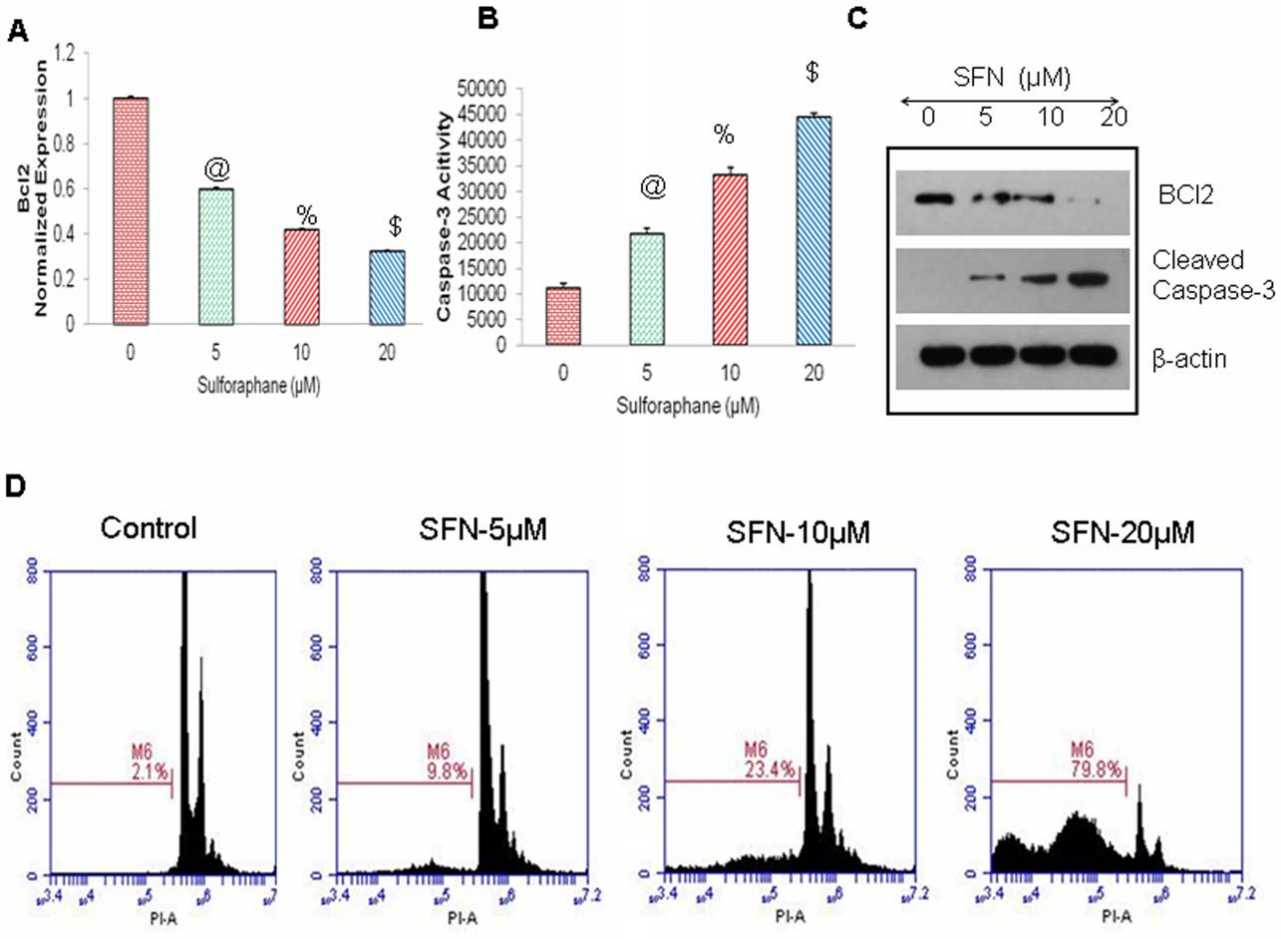
Regulation of Bcl-2 expression, caspase-3/7 activity, and apoptosis by SFN on Pancreatic CSCs. (**A**), Effects of SFN on BcL-2expression. q-RT-PCR was performed to examine the expression of BcL-2. All assays were performed in triplicate and were calculated on the basis of ΔΔ*C*t method. Data represent mean ± SD. @, % and $ = significantly different from control, P < 0.05. (**B**), Effects of SFN on caspase-3/7 activity. Pancreatic CSCs treated with SFN (0–20 µM) for 24 h, and caspase-3/7 activity was measured as per manufacturer's instructions. Data represent mean ± SD. @, %, and $ = significantly different from control, P < 0.05. (**C**), Pancreatic CSCs were treated with SFN (0–20 µM), and cell lysates were collected and Immunobloted for anti- BCL-2, cleaved Caspase 3 or β-actin antibody. (**D**), Effects of SFN on apoptosis. Pancreatic CSCs were treated with SFN (0–20 µM) for 48 h, and apoptosis was measured by PI staining using flow cytometry.

Furthermore, we have demonstrated that SFN induced apoptosis in pancreatic CSCs by inhibiting Bcl-2 expression and through the activation of caspase 3/7 (Fig. 8A–B). The inhibition of the protein expression of BCL2 and upregulation of cleaved Caspase-3, was confirmed by western blotting, as shown in (Fig. 8C). Further, flow cytometric analysis with PI staining of SFN-treated cells in (Fig. 8D), depicts that increasing doses of SFN enhanced apoptosis in pancreatic CSCs in a dose-dependent manner. These data suggest that SFN can inhibit cell proliferation and induce apoptosis in pancreatic CSCs by targeting Shh pathway and its target genes.

## Discussion

Pancreatic cancer becomes clinically apparent at late stages and it resists all forms of conventional chemotherapy and radiotherapy [1], [2]. We have recently demonstrated that CSCs share molecular characteristics with normal stem cells (SCs) and play critical roles in drug resistance and cancer metastasis. Pancreatic CSCs also demonstrate upregulation of molecules important in developmental signaling pathways, including sonic hedgehog (Shh) pathway. Uncontrolled activation of the Shh pathway has been implicated in the progression and maintenance of pancreatic adenocarcinomas [25], [26]. Pathway activation via Smo thus can occur either by Hh protein stimulation or through loss of Ptch activity, as seen in sporadic cancers or those that arise in the familial cancer predisposition syndrome, BCNS (basal cell nevus syndrome, associated with heterozygous mutation of the human Ptch gene). Activation of Shh signaling pathway is involved in the regulation of the proliferation of the pancreatic CSCs [27]. Thus, agents that inhibit Shh pathway have the potential to prevent disease progression and metastatic spread [28]. As well as drugs that selectively target CSCs offer a greater promise for cancer prevention and therapy. This project is based on the premise that SFN, a natural compound from the cruciferous vegetables, can be used for prevention and/or treatment of human pancreatic cancer. Thus, the main objective of the present study was to investigate the role of sonic hedgehog pathway in pancreatic cancer and examine the molecular mechanisms by which sulforaphane inhibits pancreatic CSC characteristics, and assess its chemopreventive/therapeutic potential against pancreatic cancer by targeting CSCs.

SFN is a naturally occurring isothiocyanate with promising chemopreventive activity [29]. Epidemiological studies have shown that people who eat cruciferous vegetables have reduced incidence of pancreatic cancer and other cancers. Test with animals have demonstrated that feeding SFN reduced the frequency, size, and number of tumors of various origins. It was well and rapidly absorbed and displayed an absolute bioavailability of 82% [30]. It is a phase 2 enzyme inducer [31], and inhibits benzo[a]pyrene-DNA and 1,6-dinitropyrene-DNA adducts formation. It acts as an antioxidant, antiproliferative, antitumor, and anti-angiogenic agent, and thus a novel candidate for chemoprevention [19], [32], [33], [34]. Some recent studies on pancreatic cancer stem cells derived from human cell lines have been reported, where they have shown that SFN can inhibit their growth through the inhibition of NFKB [35]. These studies strongly suggest that SFN can modulate the expression of genes known to play roles in the carcinogenesis process and, therefore, may be a potential agent for chemoprevention against pancreatic cancer. To the best of our knowledge this is the first study to show that sulforaphane can inhibit the growth of primary pancreatic CSCs derived from human tumors *in vitro* through the inhibition of Sonic hedgehog pathway and are thus regarded as very novel.

In the present study we have demonstrated for the first time, that cancer preventive agent SFN inhibits the expression of components of the Shh pathway in human pancreatic CSCs. SFN inhibited the expression of receptor molecule Smo, as well as effectors Gli 1 and 2, suggesting the clinical significance of Shh pathway in pancreatic cancer progression. The enforced activation of Shh or inhibition of Gli1 plus Gli2 expression by shRNA blocked the inhibitory effects of SFN, suggesting the effects of SFN are mediated through the inhibition of Shh pathway. SFN inhibits the self-renewal capacity of human pancreatic CSCs by inhibiting pluripotency maintaining factors and Shh pathway. Specifically, SFN inhibits the self-renewal capacity of pancreatic CSCs by inhibiting the expression of pluripotency maintaining transcription factors (Nanog and Oct-4), the components of Shh pathway, and induces apoptosis by inhibiting Bcl-2, Cyclin D2, and activating caspase-3 and 7. Taken together our present studies strongly suggest that SFN is a potent biologic inhibitor of human pancreatic carcinogenesis, reducing their proliferative and invasive activities. These studies raises the perspective that sulforaphane can be a beneficial agent for the prevention and/or treatment of pancreatic cancer by targeting CSCs.

Since CSCs/progenitor cells play major roles in cancer initiation, progression, recurrence and drug resistance, inhibition of CSC growth and their self-renewal capacity by sulforaphane could be significant for the management of pancreatic cancer and has been recently reviewed in several papers as a chemopreventive strategy of dietary agents for targeting cancer stem cells [36], [37]. Elucidation of the mechanism(s) for antiproliferative activity of SFN is critical to overall assessment for its potential clinical utility. Such information will not only allow rational design of sulforaphane, based strategies for prevention and/or treatment of pancreatic cancer but could also facilitate development of mechanism-driven protocols for optimal clinical effects.

## Conclusions

We have demonstrated here for the first time, that SFN inhibited self-renewal capacity of pancreatic CSCs isolated from human primary tumors and inhibits pancreatic CSC characteristics. The inhibitory effects of SFN are mediated through the inhibition of Shh pathway. SFN inhibited the expression of transcription factors (Nanog and Oct-4) which are required for maintaining stem-cell pluripotency. SFN potently eliminates the pancreatic CSCs characteristics by affecting clonogenecity, spheroid formation along with signaling involved in apoptosis resistance and proliferation. Thus, regulation of Shh signaling pathway in CSCs by non-toxic agent sulforaphane, could be considered as a novel strategy for the treatment and/or prevention of pancreatic cancer. Since aberrant Shh signaling occurs in pancreatic tumorigenesis, therapeutics that target Shh pathway may improve the outcomes of patients with pancreatic cancer by targeting CSCs and also facilitate development of mechanism-driven protocols for optimal clinical effects.

## Materials and Methods

### Reagents

Antibodies against GAPDH and Gli were purchased from Cell Signaling Technology, Inc. (Danvers, MA). SFN were purchased from LKT Laboratories, Inc. (St. Paul, MN). Enhanced chemiluminescence (ECL) Western blot detection reagents were from Amersham Life Sciences Inc. (Arlington Heights, IL). All other chemicals were purchased from Sigma-Aldrich (St Louis, MO).

### Cell culture

Human normal pancreatic stem cells (HPSC) and human pancreatic cancer stem cells (CD133^+^/CD44^+^/CD24^+^/ESA^+^) were obtained from Celprogen Inc. (San Pedro, CA). They were isolated from primary tumors and have been described previously [38]. These CSCs consist of a bulk pancreatic cancer stem cell population, and thus can not be considered as a cell line. The CSCs were cultured in DMEM supplemented with 1% N2 Supplement (Invitrogen), 2% B27 Supplement (Invitrogen), 20 ng/ml human platelet growth factor (Sigma-Aldrich), 100 ng/ml epidermal growth factor (Invitrogen) and 1% antibiotic-antimycotic (Invitrogen) at 37°C in a humidified atmosphere of 95% air and 5% CO_2_. Human pancreatic normal ductal epithelial cells (HPNE) were obtained from

Lonza, Clontetics and maintained in DMEM supplemented with growth factor bullet kit.

### Tumor spheroid assay

Spheroid forming assays were performed as described elsewhere [22], [39]. In brief, cells were plated in six-well ultralow attachment plates (Corning Inc., Corning, NY) at a density of 1,000 cells/ml in DMEM supplemented with 1% N2 Supplement (Invitrogen), 2% B27 Supplement (Invitrogen), 20 ng/ml human platelet growth factor (Sigma-Aldrich), 100 ng/ml epidermal growth factor (Invitrogen) and 1% antibiotic-antimycotic (Invitrogen) at 37°C in a humidified atmosphere of 95% air and 5% CO_2_. These cells were treated with SFN (0–20 µM). Primary spheroids were collected after 7 days and dissociated with Accutase (Innovative Cell Technologies, Inc.). The cells obtained from dissociation were sieved through a 40-µm filter, and counted by coulter counter using trypan blue dye. Secondary cultures were set up for another week, and processed as above to assess the effect of SFN on secondary spheroid formation.

### Western blot analysis

Western blots were performed as we described elsewhere [22], [39]. In brief, cells were lysed in RIPA buffer containing 1× protease inhibitor cocktail, and protein concentrations were determined using the Bradford assay (Bio-Rad, Philadelphia, PA). Proteins were separated by 12.5% SDS/PAGE and transferred to membranes (Millipore, Bedford, MA) at 55 V for 4 h at 4°C. After blocking in 5% nonfat dry milk in TBS, the membranes were incubated with primary antibodies at 1∶1,000 dilution in TBS overnight at 4°C, washed three times with TBS-Tween 20, and then incubated with secondary antibodies conjugated with horseradish peroxidase at 1∶5,000 dilution in TBS for 1 hour at room temperature. Membranes were washed again in TBS-Tween 20 for three times at room temperature. Protein bands were visualized on X-ray film using an enhanced chemiluminescence detection system.

### Caspase-3/7 Assay

Pancreatic cancer stem cells (3×10^4^ per well) were seeded in a 96-well plate with 200 µl culture medium. Approximately 16 h later, cells were treated with and without various doses of SFN. Casapse-3/7 activity was measured by a fluorometer as per manufacturer's instructions (Invitrogen).

### Apoptosis Assay

The apoptosis was determined by FACS analysis of propidium iodide (PI)-stained cells. In brief, cells were trypsinized, washed with PBS and resuspended in 200 µl PBS with 10 µl RNAase (10 mg ml/ml) and incubated at 37°C for 30 min. After incubation, 50 µl PI solution was added and cells were analyzed for apoptosis using a flow cytometry (FACSCalibur, BD Biosciences, San Jose, CA).

### Isolation of RNA

The total RNA was isolated from the pancreatic cancer stem cells using Trizol (Life Technologies) according to the manufacturer's instructions. The RNA pellets were then frozen and stored at −80°C until use.

### Evaluation of mRNA expression levels by quantitative Real Time-PCR

For the quantification of gene amplification, Real-time PCR was performed using an ABI 7300 Sequence Detection System in the presence of SYBR- Green. Briefly, RNA was isolated and reverse transcribed. cDNA reactions were amplified with QPCR SYBR Green Mix (Applied Biosystems). The following gene-specific primers were used:

Nanog (5′-ACC TAC CTA CCC CAG CCT TT -3′, 5′- CAT GCA GGA CTG CAG AGA TT -3′)

Oct4 (5′- GGA CCA GTG TCC TTT CCT CT -3′, 5′- CCA GGT TTT CTT TCC CTA GC -3′)

Smothened (5′-TCG CTA CCC TGC TGT TAT TC -3′, 5′-GAC GCA GGA CAG AGT CTC AT -3′)

Gli1 (5′-CTG GAT CGG ATA GGT GGT CT -3′, 5′- CAG AGG TTG GGA GGT AAG GA -3′)

Gli2 (5′-GCC CTT CCT GAA AAG AAG AC -3′, 5′- CAT TGG AGA AAC AGG ATT GG -3′)

Bcl-2 (5′- AGA TGG GAA CAC TGG TGG AG -3′, 5′- TCT TCA CCT CCA GGC TCA GT -3′)

PDGFRα (5′- CCA GCA GTT TCC AGT CCT AA -3′, 5′- ACA GAT TGG CAG ACC ACA TT -3′)

Cyclin D1 (5′- TTC AAA TGT GTG CAG AAG GA -3′, 5′- GGG ATG GTC TCC TTC ATC TT -3′

HK-GAPD (5′-GAG TCA ACG GAT TTG GTC GT-3′, 5′-TTG ATT TTG GAG GGA TCT CG-3′)

Target sequences were amplified at 95°C for 10 min, followed by 40 cycles of 95°C for 15 s and 60°C for 1 min. HK-GAPD was used as endogenous normalization control. All assays were performed in triplicate and were calculated on the basis of ΔΔ*C*t method. The n-fold change in mRNAs expression was determined according to the method of 2^−ΔΔCT^.

### Lentiviral reporter assay (p-GreenFire1 Lenti-Reporter)

The cop-GFP and luciferase genes were cloned downstream of Gli response element, containing four Gli binding motifs (pGreen Fire1-4xGli-mCMV-EF1-Neo; System Biosciences, Mountain View, CA) [40]. For *in vitro* assays, stably transduced pancreatic CSCs were plated at 5–10,000 cells per well in 12-well plates and treated with various doses of SFN. After incubation, CSCs were analyzed for either GFP expression by fluorometer or luciferase activity by luminometer.

### Viral production and infection

HEK 293T cells were transduced with plasmids of interest in the presence of lipofectamine. Viral supernatants were collected, mixed with PEG and concentrated by ultracentrifugation to produce virus stocks with titers of 1×10^8^ to 1×10^9^ infectious units per milliliter. Viral supernatant was collected for three days by ultracentrifugation and concentrated 100-fold. Titers were determined on HEK293T cells. Human pancreatic CSCs were transduced with a mixture of viral particles of tet-inducible Gli1 plus Gli2 lentiviral shRNA (pTRIPz, Openbiosystems), and treated with SFN as described [40]. and polybrene with two rounds of infections.

### Statistical Analysis

The mean and SD were calculated for each experimental group. Differences between groups were analyzed by one or two way ANOVA, followed by Bonferoni's multiple comparision tests using PRISM statistical software (GrafPad Software, Inc, San Diego, CA). Significant differences among groups were calculated at P<0.05.
